# Quitline Counsellors' Experiences With Vaping and Nicotine Replacement Therapy for Smoking Cessation in Australian Drug and Alcohol Services

**DOI:** 10.1111/dar.70220

**Published:** 2026-07-29

**Authors:** Jane L. Rich, Catherine J. Segan, Joshua Trigg, Ashleigh Guillaumier, Lyndell Moore, Victoria Manning, Adrian Dunlop, Joshua B. B. Garfield, Mark Daglish, Natalie Walker, Chris Bullen, Donita Baird, Dan Lubman, Coral Gartner, Amanda Baker, Billie Bonevski

**Affiliations:** ^1^ College of Health, Medicine and Wellbeing University of Newcastle Newcastle Australia; ^2^ Cancer Council Victoria Melbourne Australia; ^3^ Melbourne School of Population and Global Health University of Melbourne Melbourne Australia; ^4^ Flinders Health and Medical Research Institute, College of Medicine and Public Health Flinders University Adelaide Australia; ^5^ Monash Addiction Research Centre, Eastern Health Clinical School Monash University Melbourne Australia; ^6^ Turning Point, Eastern Health Melbourne Australia; ^7^ Drug and Alcohol Clinical Service, Hunter New England Area Health Newcastle Australia; ^8^ Faculty of Medicine The University of Queensland, and Royal Brisbane and Women's Hospital Brisbane Australia; ^9^ School of Population Health, Faculty of Medical and Health Sciences University of Auckland Auckland New Zealand; ^10^ School of Public Health, Faculty of Medicine The University of Queensland Brisbane Australia; ^11^ National Drug and Alcohol Research Centre UNSW Sydney Sydney Australia

**Keywords:** aftercare, counsellors, nicotine replacement therapy, smoking cessation, workforce

## Abstract

**Introduction:**

In Australia, people undergoing inpatient treatment for alcohol and other drug (AOD) use disorders have a high prevalence of tobacco smoking. Smoking cessation support is routinely offered to clients within inpatient withdrawal settings, but continued support post‐discharge typically is not. The current study qualitatively explores attitudes and experiences of Quitline counsellors involved in a two‐armed trial for clients with provision of 12 weeks of nicotine vaping products (NVPs) or combination nicotine replacement therapy (cNRT).

**Methods:**

Two focus groups of Quitline counsellors (six per group) explored how they felt about training received, their beliefs and attitudes about NVPs and cNRT for smoking cessation for AOD‐use population and their wider experience supporting AOD clients. Data were inductively coded.

**Results:**

Six themes were identified: (i) Counsellor Readiness and Professional Adaptation to Emerging Cessation Tools; (ii) Addressing Complexity: Behavioural Support in Multi‐Substance Recovery Contexts; (iii) Client Preferences and Product Engagement in Cessation Support; (iv) Continuity of Care: Bridging Residential Treatment and Community Support; (v) Enablers of Quality Practice: Training, Resources and Organisational Support; and (vi) Service Integration and Boundary Management in Specialised Populations.

**Discussion and Conclusions:**

The agility of Quitline services models their potential to partner with clinical research teams and AOD services to build the evidence‐base around the effectiveness of reduced‐harm nicotine products for people with complex needs. The Victorian Quitline partnership with the NEAT trial produced a comprehensive service model assisting people seeking AOD treatment to quit smoking that highlights the workforce as a modifiable factor by involving staff in evidence‐building adaptability.

## 1 Introduction

In Australia, people accessing inpatient alcohol and other drug (AOD) use treatment services are seven times more likely to smoke tobacco daily, compared to the general Australian population (84% vs. 12%, respectively) [1]. Consequently, they face a greater risk of tobacco‐related health issues and are more likely to die from smoking‐related illnesses than from issues relating to their other substance use [2, 3]. Best practice treatments for tobacco smoking cessation in Australia combine multi‐session behavioural counselling (such as that delivered by Quitline) with smoking cessation pharmacotherapy [4]. Implementation of best practices and timely treatment that meets the needs of AOD treatment clients is needed to optimise smoking cessation outcomes in this population.

In Australia, inpatient AOD withdrawal programs are predominantly smoke‐free and provide free combination nicotine replacement therapy (cNRT, i.e., nicotine patch plus a faster‐acting oral form of cNRT) to clients while on‐site, to alleviate nicotine withdrawal symptoms. However, AOD services do not typically provide further cNRT on discharge, resulting in rapid return to smoking among most clients [5].

The Australian ‘NicotinE As Treatment’ (NEAT) trial [6] sought to reduce the rate of smoking relapse following discharge from inpatient AOD services, through testing a novel service delivery model that offered: (i) Quitline behavioural counselling initiated during the period of enforced smoking abstinence to help patients reflect on their experience of being smokefree and to decide whether they wanted to continue use of nicotine products post‐discharge (specifically, participants were randomised and offered 12 weeks supply of either nicotine vaping products (NVP) or cNRT post‐discharge); and (ii) Quitline counselling and psychoeducation post‐discharge to help clients utilise and further develop skills required to stay abstinent or to minimise tobacco smoking while navigating the rapid increase in triggers typically experienced following re‐entry into their regular social world.

Multi‐session behavioural counselling for smoking cessation delivered via telephone is effective and its accessibility, convenience and personalised approach makes it a valuable tool to help people quit smoking and maintain long‐term abstinence [7]. These features have proven useful for those who face access barriers, such as geographic distance, transport limitations or social stigma associated with accessing in‐person counselling. In this study, Quitline counselling aimed to build motivation and confidence to maintain smoking abstinence via psychoeducation and skills building, including identifying smoking triggers, exploring alternative strategies to remain abstinent and helping people to optimise their use of pharmacotherapies.

For the NEAT trial [6] Quitline counsellors were trained to provide cessation support to participants in smokefree inpatient withdrawal programs. The counsellors were trained by clinical psychologists (D.B., A.B.) and behavioural scientists (B.B., C.S.) from the research team. The counselling goals were to: (i) instil optimism and confidence regarding addressing tobacco smoking; (ii) encourage clients to use cNRT or NVP instead of smoking when they left the residential setting; and (iii) try to reduce psychological dependence on cigarettes by finding alternate coping strategies. The first counselling call to participants was scheduled while in residential care, with post‐discharge calls focusing on relapse prevention. Zhu's relapse prevention call schedule [8] was followed, with calls planned for Days 1, 3, 7, 14 and 28 post‐discharge. The total number and timing of calls was tailored to client need and smoking status (i.e., more frequent calls were scheduled around relapse crises/quit attempts) with a maximum of 15 calls over 12 weeks. Participants were sent a text message prior to being called to encourage answering calls from a private number (Quitline).

Participant recruitment for the NEAT trial [6] commenced in December 2020 and ended in June 2022. We undertook a qualitative study to explore the attitudes and experiences of the Quitline counsellors who delivered tobacco smoking cessation counselling to NEAT participants. Key aims were to explore counsellor attitudes towards the different pharmacological products on offer (cNRT and NVP), experiences of counselling participants using these products and their experiences in providing smoking cessation support to AOD clients.

## 2 Methods

Human research ethics approval was granted by the Hunter New England Area Health (REGIS: 2019/ETH10554), University of Newcastle Human Research Ethics Committee (H‐2019‐0358), as well as Cancer Council Victoria Human Research Ethics Committee (HREC2001). The study follows COREQ reporting standards [9]. At the time of the study, Victorian Quitline counsellors were comprehensively trained in cNRT, but not NVPs as they are not approved for cessation by Australia's Therapeutic Goods Administration. For this trial [6], Quitline counsellors were trained by clinical psychologists (D.B., A.B.) and behavioural scientists (B.B., C.S.) from the research team in providing counselling to participants in both conditions (cNRT or NVP) of NEAT. Counsellors recruited for the focus group were involved in the provision of counselling to trial participants for at least 1 year. Australian Quitline counsellors are required to be non‐smokers—former smokers are recommended to have quit smoking for at least 6 months. The trial protocol was registered with the Australian and New Zealand Clinical Trials Registry (12619001787178) and protocol published via preprint [10], with main outcomes published in 2025 [6].

### 2.1 Design and Eligibility

Two focus groups were undertaken in October 2022; discussions were designed to explore the attitudes and experiences of Quitline counsellors who delivered tobacco smoking cessation counselling to participants in the randomised controlled trial described above. To be eligible for the study, Quitline counsellors had to have provided counselling to trial participants for at least 1 year.

### 2.2 Procedures

Eligible Quitline counsellors were sent an email invitation to participate in a confidential 1‐h online focus group about their experiences in the NEAT trial [6]. The invitation was emailed to counsellors by a member of the research team (who was also employed by the Cancer Council Victoria [C.S.]). Consent forms and participant demographics were then provided to the lead author (J.L.R.), who subsequently organised and conducted the focus groups. Counsellors were reminded that their involvement was voluntary, confidential and that they could withdraw from the study prior to the focus group commencing.

The focus groups were facilitated by the lead author (J.L.R.) and conducted, recorded and transcribed using Microsoft Teams (version 1.6.00.28554). The focus groups occurred during the counsellors' scheduled work shift and participants were offered a summary of findings on study completion. Participating Quitline counsellors were asked to focus on their experience in the clinical trial, including the training they received, the support they provided to clients, their attitudes regarding the reduced harm nicotine products offered (cNRT, NVP) and their experience providing support to people with AOD use disorders to quit smoking.

The full focus group schedule (Supporting Information File A) covered counsellors' views on: (i) training in cNRT and NVP support provision to clients; (ii) use and adequacy of the counselling manual for support provision; (iii) clients' responses regarding psychoeducation, quitting self‐efficacy, skills and strategy building; (iv) beliefs and expectations about cNRT and NVP effectiveness for clients; (v) experience of supporting clients of AOD services compared to other Quitline clients; (vi) usefulness of NVP compared to cNRT for client tobacco smoking cessation; and (vii) any unanticipated aspects of providing Quitline support for clients of AOD services.

### 2.3 Analyses

Focus groups were transcribed using Microsoft Teams' automated transcription function and transcripts were checked and corrected against the video recordings by the lead author (J.L.R.). Data were analysed using a coding approach to thematic analysis [11], which combines the structured, predominantly deductive coding suited to focused evaluation questions with flexibility to capture unanticipated patterns in the data. This approach was selected because the analysis was oriented to the NEAT trial's specific evaluation aims: to examine Quitline counsellors' attitudes, experiences and perceived barriers in providing counselling to trial participants using either cNRT or NVP.

An initial coding framework was developed from the domains of the focus group guide. J.L.R. coded all transcripts in NVivo (v12, QSR International), applying this framework while remaining open to inductive codes for content falling outside it. Codes and candidate themes were reviewed and refined through discussion between J.L.R. and L.M.; these discussions served to interrogate and develop the analysis rather than to establish coding agreement, consistent with the qualitative orientation of the approach. Related codes were grouped into themes addressing the evaluation questions and theme development continued iteratively until the themes coherently captured patterning across the dataset.

As a qualitative researcher, the lead author (J.L.R.) was acutely aware of her position in coding and analysing the data. J.L.R. was also the Trial Coordinator for NEAT and had been involved with the study since its inception. J.L.R. was aware that she brought to the analysis her own bias and values regarding this project, namely the intention for those that wish to cease smoking to have the opportunity and support required. J.L.R. did not have strong views regarding the smoking cessation options available to participants but to support [11] and provide a safe trial—positioning herself in this way, being open and transparent about one's roles, values and perspectives enables more credibility and richness to the interpretation of qualitative findings.

Following all manual analysis (J.L.R.) and team discussions, the results were written up by J.L.R. Claude AI (Anthropic, version 4.5) was then used as a collaborative tool to refine the presentation of theme titles, reviewed and minor changes made for appropriateness by J.L.R. All thematic coding, interpretation and substantive analytical decisions were conducted by the research team.

## 3 Results

Twenty‐three Quitline counsellors were involved in the NEAT trial. At the time of recruitment for the focus groups, eight of the counsellors had left Quitline. Twelve of the 15 invited counsellors participated in a focus group. Two online focus groups with six participants per group were conducted on the 18 October 2022.

The median age of focus group participants was 40 years (range 36–63 years), eight of the 12 counsellors identified as female and four identified as male. All counsellors reported being employed part‐time, with a median duration of 9 years as a Quitline counsellor (range 1–19 years). Ten counsellors reported never having used NVPs and two reported ever‐use, ‘even a puff’, of NVPs.

Six themes captured counsellors' experiences delivering cessation support to people in addiction treatment, revealing broader insights for tobacco treatment services working with complex populations. These included:

1. Counsellor readiness and professional adaptation to emerging cessation tools.
2. Addressing complexity: behavioural support in multi‐substance recovery contexts
3. Client preferences and product engagement in cessation support.
4. Continuity of care: bridging residential treatment and community support.
5. Enablers of quality practice: training, resources and organisational support.
6. Service integration and boundary management in specialised populations.

### 3.1 Counsellor Readiness and Professional Adaptation to Emerging Cessation Tools

This theme examines the service capacity and ability to adopt new interventions, highlighting the balance between evidence‐based practice and innovation adoption. Counsellors shared their various attitudes and feelings regarding NVP use.

Overall, the Quitline counsellors were open to the role that NVPs may play in enabling smoking cessation among this population. However, the counsellors had reservations regarding the use of these products due to insufficient efficacy and safety evidence. Nevertheless, the use of NVPs in the context of the trial was viewed as appropriate, given its focus on generating evidence regarding the effectiveness of NVPs as a smoking cessation aid within this population. The counsellors felt well‐supported in their role, knowing they were contributing to important research and cited the study protocols, counselling manual and the research team as supporting their counselling. Their expertise and comfort in supporting participants with the use of NVPs increased over the study period and they described feeling inspired by the courage and efforts of trial participants.

I honestly didn't form an opinion about it (NVPs) to be honest because it was a research trial. So, you know that's what we were aiming to find out—Participant 7

Cautiousness … Around, you know, vaping and I suppose, maybe with the wider Quitline call(er) population even more caution. But, perhaps I'm a bit more enthusiastic about the vape and the success that we're having with these new clients here—Participant 4

I felt a little bit at that time at a loss about the vapes … It was contradictory in the sense that here we are providing these products [without evidence], yet we know everything about cNRT—Participant 2

### 3.2 Addressing Complexity: Behavioural Support in Multi‐Substance Recovery Contexts

This theme highlights the unique challenges of delivering cessation support alongside addiction treatment, making it relevant for any integrated care setting dealing with multi‐substance use. A core component of Quitline counselling consists of working with clients to make behavioural changes to cease or reduce tobacco smoking. Understanding behavioural habits, triggers, emotions and frame of mind is critical to the smoking cessation journey. This theme included subthemes such as skill building, education, ‘the real work’, therapy and beliefs.

Quitline counsellors were well equipped to deliver psychoeducation and felt that their clients welcomed such discussions. However, the counsellors acknowledged that this population faced many challenges and often experienced withdrawal symptoms related to multiple substances, making delivery of information difficult.

It was almost a given, with the [AOD service] clients that there was another drug [in use/withdrawing] which you're very conscious of the fact that tobacco is very intertwined with the use of the other drugs—Participant 11

I would have had a bit more input about behavioural stuff, but my sense was for quite a lot of clients, they couldn't go there yet. They, you know, there [were] a lot of [cognitive] barriers for them in terms of being able to have that insight—Participant 10

It is quite difficult to start, until you, until they, believe actually they have it within themselves to be able to work through the process, trusting the process [of self‐reflection]—Participant 6

Overall, the Quitline counsellors were very enthusiastic and confident in exploring behaviour change with the NEAT participants and believed that this was crucial to smoking cessation. Some counsellors acknowledged the skills the clients had already gained through the inpatient treatment program. However, some counsellors observed that in cases where clients simply replaced their smoking with vaping behaviour, deeper change was more difficult to explore.

I'll use the vape. I'll use the vape. I'll use the vape. Rather than working on the behavioural side of things—Participant 2

Perhaps there was more, you know, focus perhaps, or willingness to focus on those other areas of developing skills around the behavioural side of quitting with the cNRT group … perhaps a more balanced way because there wasn't such a belief that the vapes are magic—Participant 5

We were able to acknowledge their expertise, because they had just done it [completed their inpatient stay smoke‐free]—Participant 9

### 3.3 Client Preferences and Product Engagement in Cessation Support

This result highlights the broader issue of matching cessation tools to client needs and expectations. The focus groups discussed client preferences for cessation tools, barriers, costs and benefits as well as the user experience. These elements were brought together under the theme of Type of Nicotine Products: NVP versus cNRT. Counsellors explained their experience that, undoubtedly, participants in the NEAT trial were hopeful they would be allocated the NVP and would often be upset and disappointed if they were allocated to the cNRT condition.

Counsellors were able to educate participants on the benefits of receiving either cNRT or the NVP and communicated that both options assisted smoking cessation. There was discussion around the safe use of cNRT and NVP, as well as the beliefs that NEAT trial participants held about the qualities of the products, including nicotine dosage.

It's good they got access to a few cNRT products. Because then they could find their best fit as well, rather than not having the money and going ‘let's try the gum then, I don't really like this’ and then having to wait until they're paid next to try something else, whereas this way they could kind of go ‘this works for me, and this doesn't—Participant 5

They enjoyed the vape more, so in a way I felt like that was a good thing, because if they're using a product designed to help them quit, then we hope it will [work]—Participant 1

Often with the cNRT people don't like the taste of the oral [formulations] and they just don't use it. Well, you can't help them if they're not using it enough at least with a vape, they used it even if they may have overused it!—Participant 12

### 3.4 Continuity of Care: Bridging Residential Treatment and Community Support

This theme explored the care transition challenge, a universal experience across addiction services, highlighting the importance of warm handoffs and sustained engagement between residential treatment services and community‐based support services including Quitline.

Communication was an important component to both the operational side of the NEAT trial, as well as the therapeutic role of Quitline for participants. Communication with clients and the recruitment sites included: communication with participants, communication with AOD services, and feedback and input from the trial coordinating centre. The counsellors shared their insights and in general expressed their admiration for participants, whom they perceived as receptive to their calls and had a willingness to engage with Quitline.

The counsellors also expressed how critical it was to make initial contact with the participants during their stay at the AOD treatment facility, prior to discharge. However, several challenges were observed. These included communication with AOD facilities at times, COVID‐19 restrictions and the unpredictable environments and lives that the participants were returning to.

Most of the time they [participants] were pretty good [communicating with Quitline], they kind of made that decision to do it [quit], they made that decision to be part of the study. So, they wanted to follow it through particularly if they were having a good experience with the products that were given—Participant 3

I have a sense that they felt obligated [to communicate with Quitline] because they were getting free products, but they always picked up, were engaged, generally speaking—Participant 9

Sometimes it was hard to get through to the facility, depending on when you rang, but in general it was really good—Participant 5

If we could make that contact before they left because it's set up a relationship and then I felt they were, you know, more likely to continue that relationship with Quitline afterwards—Participant 7

### 3.5 Enablers of Quality Practice: Training, Resources and Organisational Support

Exploring what enables counsellors to deliver effective support in any implementation or service is critical. Quitline counsellors felt supported by the trial coordinating centre, training, materials and communicated that they were more inclined to discuss vaping‐related issues as a result of the training resources provided. Counsellors reported that they often referred to the manual and ‘cheat sheets’ provided. However, the significant delay between the conclusion of training and start of the trial (due to COVID‐19 lockdowns) was identified as a limitation and counsellors indicated that a brief refresher prior to the start of the trial would have been useful.

[The training] de‐escalated any anxiety we might have had; the training was excellent—Participant 8

I constantly refer to the manual because we don't deal with [NEAT] clients all the time, so you do forget very quickly, you're back on mainstream clients and then you have a [NEAT] client, so I always have the manual next to me—Participant 4

I feel like we're already well equipped to do it because we were already trained in cNRT and those products, the part we didn't know much about was the vape but I remember the presentation was thorough and I also felt supported by the fact that we were explicitly told if the participants had issues with the vape that they could call the study and we didn't have to answer mechanical questions about the vape so that worked quite well for me—Participant 8

### 3.6 Service Integration and Boundary Management in Specialised Populations

This theme explored the broader challenge of scope, managing complexity and integrating smoking cessation within comprehensive care, which is applicable to any specialist service working with a targeted population. All counsellors felt that the Quitline service was appropriate, well‐received and useful for NEAT participants. However, clear communication of Quitline's focus on smoking cessation and abstinence rather than long‐term substitution was required, along with the ability to refer participants to other service providers for assistance with other drug‐related issues. One counsellor shared their early cynicism and subsequent shift in views of just how useful Quitline may be for this population.

I've only ever had negative experiences in my life of people with substance abuse issues, so personally, I was [a] sceptic. I wasn't that looking forward to taking part in the study to be honest with you and I wasn't sure if this group of people specifically would like it [Quitline] … but in doing this study because of the level of engagement of [NEAT] client[s], I ended up feeling that Quitline did suit this group and a big percentage of them found our support helpful, so that was good for me personally because I felt ‘How am I gonna help this group? These people are so complex and I'm getting triggered’ it was a good experience and I felt Quitline was very, very successful—Participant 8

I think it's good how we set the boundaries from first call to say you know, we're not AOD counsellors. We're here to support you [with] smoking cessation—Participant 2

You know for some, it's their first time they've actually quit smoking [inpatient withdrawal program], so this is gold. You know they have a chance now. Even if they don't sustain it. Giving them an opportunity to link into Quitline at this time, [is] so important—Participant 10

## 4 Discussion

The analysis provides findings regarding the attitudes and approaches of Quitline counsellors supporting smoking cessation among individuals receiving AOD treatment. Analysis highlights how training and innovation can transform build confidence in the workforce. The counsellors demonstrated a balance of optimism and caution, driven by a desire to explore NVPs' potential as a smoking cessation aid while acknowledging the limited evidence‐base to support their use for this purpose. Counsellors' engagement was strengthened by their involvement in meaningful research, that is, a clinical trial that would inform practice and policy. The counsellors' expertise in applying progressive behavioural strategies [12] highlights their adaptability and commitment to individualised care. Effective communication, training and resources were essential components for their support and the Quitline counsellors felt their service was beneficial, despite some initial scepticism.

These findings emphasise the importance of comprehensive models of care in addressing the gap in smoking cessation services. There is a lack of systematic integration between residential treatment and community‐based cessation support. Health systems looking to reduce tobacco‐related disparities should prioritise similar cross‐service partnerships, especially for those facing multiple barriers to conventional smoking cessation support.

The NEAT trial [10] and the preceding pilot study [13], were the first opportunities for Quitline counsellors to provide smoking cessation counselling to people prescribed nicotine vaping products. On 1 October 2021 (after trial recruitment had begun), the Australian medicines regulator clarified the classification of NVPs as a prescription only medicine and implemented a streamlined process to facilitate doctors prescribing NVPs as a smoking cessation aid. This regulation change formalised the use of NVPs as a second‐line treatment for smoking cessation in people where first‐line treatment (pharmacotherapies approved by the regulator plus behavioural counselling) has been unsuccessful [12]. In preparation for the 2021 regulatory changes, Quitline introduced two counselling protocols: ‘NVPs for smoking cessation’ and ‘Stopping vaping’ for people seeking help with vaping cessation, based on the clinical guidelines from the Royal Australian College of General Practitioners [12]. Quitline offers support to people trying to quit smoking using NVPs, whether they are prescribed or not. This regulatory change and the findings from this study, highlight the agility of Quitline in responding to the needs of those attempting to switch away from tobacco use. Quitline encourages clients to see NVP use as a short‐term option for smoking cessation, given that they are not risk‐free, with this risk balanced against the priority of preventing smoking relapse.

This research highlights the important role of community‐based collaborations in smoking cessation clinical trials. Smoking cessation models require partnership, integration and embedded support to best address people's needs, that is, in this case connecting clients whilst in smokefree AOD withdrawal programs to Quitline. This qualitative research has highlighted the flexibility and responsiveness of services such as Quitline to continue the front‐line cessation work that they do, especially when policy and regulatory positions change. Quitline counsellors have not only been responsive to this change but reflective too, valuing their contribution to building the evidence‐base and appreciating that while NVPs may help some clients to quit smoking, they may also help people who have not previously been interested in quitting.

There are limitations to this research, including the fact that not all counsellors involved in NEAT participated in the focus groups. Counsellor views that were not captured in this data collection may have provided additional insights. It is also acknowledged how challenging it is to monitor and manage the growing use of illicit NVPs in Australia and that these findings relate directly to a process that was undertaken as part of a clinical trial whereby NVPs were provided in a therapeutic context to assist tobacco cessation. However, these limitations do not diminish the study's central contribution: identifying the workforce development, service integration and clinical practice requirements for delivering equitable smoking cessation support to a population that experiences profound tobacco‐related harm yet is frequently excluded from cessation initiatives.

## 5 Conclusion

In working directly with the AOD population this study has shown that specialised NVP training would be beneficial. The partnership of Quitline with the NEAT trial has produced a comprehensive service model for assisting people using withdrawal services to stay smokefree following discharge, building capacity to service those with the greatest need.

The counsellors in this study demonstrated that with appropriate preparation and support, frontline services can adapt to meet emerging challenges while maintaining their commitment to client‐centred, evidence‐informed care. Their experience offers a roadmap for the broader cessation sector facing similar demands for adaptation and inclusion.

## Author Contributions

**Jane L. Rich:** conceptualisation, methodology, formal analysis, data curation, writing – original draft, writing – review and editing, project administration. **Catherine J. Segan:** conceptualisation, methodology, resources, data curation, writing – original draft, writing – review and editing, supervision, project administration, funding acquisition. **Joshua Trigg:** conceptualisation, formal analysis, data curation, writing – original draft, writing – review and editing. **Ashleigh Guillaumier:** writing – original draft, writing – review and editing. **Lyndell Moore:** formal analysis, writing – review and editing, project administration. **Victoria Manning:** writing – review and editing, funding acquisition. **Adrian Dunlop:** writing – review and editing, funding acquisition. **Mark Daglish:** writing – review and editing, funding aquisition. **Joshua B. B. Garfield:** writing – review and editing. **Natalie Walker:** writing – review and editing, funding acquisition. **Chris Bullen:** writing – review and editing, funding acquisition. **Donita Baird:** methodology, resources, writing – review and editing, funding acquisition. **Dan Lubman:** writing – review and editing, funding acquisition. **Coral Gartner:** writing – review and editing, funding acquisition. **Amanda Baker:** writing – review and editing, funding acquisition. **Billie Bonevski:** conceptualisation, methodology, resources, data curation, writing – original draft, writing – review and editing, supervision, funding acquisition.

## Funding

This study was funded by the Australian National Health and Medical Research Council (Grant G1800272).

## Conflicts of Interest

The authors declare no conflicts of interest.

## Supporting information


**File A.** Demographic survey and focus group schedule.

## Data Availability

The data that support the findings of this study are available from the corresponding author upon reasonable request.
