# Supplementary material for: Quitline Counsellors' Experiences With Vaping and Nicotine Replacement Therapy for Smoking Cessation in Australian Drug and Alcohol Services
Source: Drug Alcohol Rev. 2026 Jul 29;45(6):e70220. doi: 10.1111/dar.70220 (PMC13420958; doi:10.1111/dar.70220)
Supplement: Supplementary file 1 — File A. Demographic survey and focus group schedule. [file DAR-45-0-s001.docx]

**File A – Demographic survey and focus group schedule**

**Introduction**

Hi, my name is ______________. I’m from the Project NEAT study and was hoping to ask you a few questions about your involvement in the trial, as well as a few questions about your demographics. If you’re interested, the interview/ focus group can be completed over the phone/ or in person and will take about 20 to 30 minutes. The interview/ focus group will be audio-recorded and transcribed word for word. We’ll be interviewing other people involved in the trial to collect a wide spread of experiences and we will use that information to guide future practice and to write up and share in scientific journals and conferences. All the information is confidential, which means that you won’t be identifiable. We might quote you when we share this information, but we wouldn’t use your name, we would just use generic information to describe you, like your age and gender. Are you happy to go ahead with the interview?

**Demographic Survey**

1. **What is your main role/ job title at Quitline?**

| Quit Specialist | 1 |
| --- | --- |
| Aboriginal/Torres Strait Islander Quit Specialist | 2 |

1. **On what basis do you work at Quitline?**

| Full-time | 1 |
| --- | --- |
| Part-time | 2 |

1. **How long have you worked at Quitline?**

| years |
| --- |

1. **Do you identify yourself as:**

| Male | 1 |
| --- | --- |
| Female | 2 |
| Other | 3 |

1. **Do you identify yourself as:**

| Aboriginal | 1 |
| --- | --- |
| Torres Strait Islander | 2 |
| Aboriginal and Torres Strait Islander | 3 |
| Neither | 4 |

1. **What is your age?**

| years |
| --- |

1. **Have you ever tried a nicotine vaping product?**

| Yes | 1 |
| --- | --- |
| No | 2 |

1. **Do you currently use a nicotine vaping product?**

| Yes | 1 |
| --- | --- |
| No | 2 |

**Focus Group Schedule**

(not bold text = prompts)

- **Was there sufficient training to deliver Quitline counselling to AOD clients using NVP (e-cigarette) /NRT as part of the NEAT trial?**
  - How confident did you feel in delivering Quitline counselling to these clients?
  - What information did you feel was important for you to know to best support these clients quit smoking?
  - What suggestions do you have for improving the training?
- **How useful was the counselling protocol and the tip sheets provided as part of this trial?**
  - Did you read them?
  - What was most useful?
  - Did you refer back to them at all when reflecting on your calls with AOD clients?
  - Any ideas for improving the counselling protocol or tip sheets?
- **Did you find it easy to contact clients?**
  - Did clients pick up the phone and talk to you about quitting?
  - Did they request no further contact, and if so, what was their reason?
  - What could be improved to make it easier for AOD clients to engage with Quitline?
- **How did clients respond to assessment/monitoring of their smoking habits, and quitting moods and experiences?**
  - How could assessment/monitoring be improved?
- **How did clients respond to psychoeducation about quitting?**
  - Did you feel that you had sufficient information to provide to clients?
  - Was the information appropriate for clients’ intellectual capacity?
  - Was the information relevant to clients’ cessation needs?
  - What information do you believe clients found most useful?
  - How could psychoeducation be improved to better suit clients?
- **How did clients respond to skills building information?**
  - How could skills building information be improved?
- **How did clients respond to self-efficacy assessment and development?**
  - How could self-efficacy assessment and development be improved?
- **Did you find it easy to liaise with AOD services?**
  - Did they give you information that you needed to contact clients effectively?
  - Did you have to contact them for any further information?
  - How could communication between Quitline and AOD services be improved?
- **Did you believe that NVP would help these clients quit smoking? Why?**
  - Has your belief changed since the trial began?
  - How has it changed/stayed the same?
  - **Did you believe thatcNRT would help these clients quit smoking?** Why?
  - Has your belief changed since the trial began?
  - How has it changed/stayed the same?
- **Were many clients disappointed that they hadn’t been assigned to receive the other type of nicotine product? i.e.cNRT or NVP?**
  - **Did you believe Quitline would help these clients quit smoking?** Why?
  - Has your belief changed since the trial began?
  - How has it changed/stayed the same?
- **How was smoking cessation support provision different for AOD clients compared with clients without AOD use?**
  - Query content differences, number of calls, time per call, engagement, etc.
- **Did you find you had enough time to provide AOD clients with tailored support?**
- **Could anything be improved to help AOD clients in this trial to use NVP/NRT and engage with Quitline to help them quit smoking?**
- **If you came across an AOD client who was using a NVP to quit smoking would you encourage them to continue using the NVP?**
- **Would you encourage AOD clients to use Quitline to help them quit smoking in the future?**
- **Do you think all clients in withdrawal units should be offered a call from Quitline while they are at the withdrawal unit?**
- **Did you find that many participants were having difficulty using their NVP/NRT and that you needed to direct them to contact the research team?**
- **Did you feel supported throughout your involvement in the trial?**
  - Did you have access to useful support to deliver the trial?
  - Did you feel comfortable accessing support if it was required?
- **Did you have ongoing contact with the research team to help troubleshoot any issues the participant was having?**
- What kind of issues were referred back to the research team?
- Did you find this process easy and effective?
- What kind of issues were resolved in-house with the Quitline manager?
